# Biological and therapeutic role of LSD1 in Alzheimer’s diseases

**DOI:** 10.3389/fphar.2022.1020556

**Published:** 2022-10-25

**Authors:** Yu Li, Yuanyuan Zhao, Xiaona Li, Liuqun Zhai, Hua Zheng, Ying Yan, Qiang Fu, Jinlian Ma, Haier Fu, Zhenqiang Zhang, Zhonghua Li

**Affiliations:** ^1^ Department of Pharmacy, Yellow River Central Hospital of Yellow River Conservancy Commission, Zhengzhou, China; ^2^ Department of Pharmacy, The Third Affiliated Hospital of Zhengzhou University, Zhengzhou, China; ^3^ Academy of Chinese Medical Sciences, Henan University of Chinese Medicine, Zhengzhou, China

**Keywords:** alzheimer’s disease, epigenetics, demethylase, LSD1, inhibitors

## Abstract

Alzheimer’s disease (AD) is a common chronic neurodegenerative disease characterized by cognitive learning and memory impairments, however, current treatments only provide symptomatic relief. Lysine-specific demethylase 1 (LSD1), regulating the homeostasis of histone methylation, plays an important role in the pathogenesis of many neurodegenerative disorders. LSD1 functions in regulating gene expression *via* transcriptional repression or activation, and is involved in initiation and progression of AD. Pharmacological inhibition of LSD1 has shown promising therapeutic benefits for AD treatment. In this review, we attempt to elaborate on the role of LSD1 in some aspects of AD including neuroinflammation, autophagy, neurotransmitters, ferroptosis, tau protein, as well as LSD1 inhibitors under clinical assessments for AD treatment.

## 1 Introduction

Alzheimer’s disease (AD) is a chronic and complex neurodegenerative disease, most often associated with cognitive function disorders including memory decline, learning deficits, psychiatric disorders, and spatial disorientation, etc., AD is most popular in people over the age of 65, and the risk of AD increases with age. With the aggravation of the aging problem worldwide, AD population is estimated to reach more than 131 million by 2050 barring the development of effective treatment to prevent AD (Cummings et al., 2016). Pathologically, AD is a complex and multifactorial disease, with a number of factors accounting for the prognosis of AD, such as chronic inflammation, synaptic dysfunction, amyloid plaques accumulation, neurofibrillary tangles, and neuronal death (Long and Holtzman, 2019; Ju and Tam, 2022). Over a long period of time, much attention has been paid to AD treatments based on the classical amyloid cascade and tau aggregation hypothesis. However, despite considerable research efforts to tackle these issues, there has been a paucity of effective treatment options for AD patients, and over 200 AD investigational programs have failed over the past decades (Yiannopoulou et al., 2019; Majernikova et al., 2021). Therefore, there are urgent needs for effective treatments for this condition.

In recent years, epigenetic mechanisms have emerged as an important player in central nervous system (CNS) disorders (Coppede, 2021). Epigenetic regulations shape the phenotype without altering the genotype, and mainly include DNA methylation, histone modifications, chromatin remodeling, nucleosome positioning and non-coding RNAs (Stoccoro and Copped, 2018). Histone methylation represents one of the best characterized post-transcriptional modifications on histone tail residues, and is associated with neural functions related to cognitive abilities (Bannister and Kouzarides, 2011; Collins et al., 2019; Lopez-Tobon et., 2020). Increasing studies suggest that histone methylations play essential roles in neuronal differentiation and plasticity, as well as in memory and learning process, while impaired regulation of histone methylation are tightly associated with neurodegenerative disorders (Fischer et al., 2007; Herre and Korb, 2019). Thus, targeting histone methylation has been proposed to be a novel therapeutic strategy for AD (Lin et al., 2019; Zheng et al., 2019).

Lysine methyltransferases and demethylases are responsible for catalyzing the process of N-methylation and N-demethylation of histone lysine, respectively (Kaniskan et al., 2018). Unlike histone acetylation, phosphorylation and ubiquitination, which were identified as a dynamic process, histone methylation has historically been considered as an irreversible mark until the discovery of lysine specific demethylase 1 (LSD1) in 2004 (Shi et al., 2004). Through maintaining a proper situation of histone methylation, LSD1 makes a broad scope of regulatory effects on histone or nonhistone proteins (Huang et al., 2007; Wang et al., 2009; Ciccone et al., 2009; Kontaki and Taliandis, 2010). Several lines of research have revealed that, by forming distinct protein complexes, LSD1 contributes to various biological processes including cell proliferation, stem cell biology, autophagy, metabolism and neural development (Wang et al., 2007; Stefano et al., 2016; Maes et al., 2015). The aberrant function of LSD1 has been found to be able to stimulate many negative biological processes such as tumor initiation and progression, and is closely connected with various malignant tumors, especially lung cancer and leukemia (Wang et al., 2009; Magerl et al., 2010; Lokken and Zeleznik-Le, 2012). Either genetic depletion of LSD1 or pharmacological inhibition has been shown to suppress the tumor growth, and therefore LSD1 is becoming a promising therapeutic target for cancer treatment (Schmitt et al., 2013).

In CNS, LSD1 plays a crucial regulatory role in the maintenance of pluripotency and in specification of neuronal commitment of multipotent cells, which is proposed to be an important hallmark of the situation and progression of many neurodegenerative disorders such as AD (Maiques-Diaz et al., 2018). There are currently no findings about genetic disorders affecting brain development or function caused by LSD1 mutations (Maes et al., 2015). However, by forming distinct protein complexes probably affected by mutation or epigenetic modification, modulation of LSD1 activity is proposed to be able to improve the symptoms of neurodegenerative disease. For instance, the repressor complex of LSD1-REST-CoREST-HDAC1/2 functions primarily in controlling developmental program and modulating neuronal morphology in CNS diseases (Toffolo et al., 2014; Saez et al., 2015). Christopher et al. (2017) reported that inducible depletion of LSD1 in mice led to paralysis along with neurodegeneration of hippocampus and cortex, causing learning and memory deficits. Mechanistically, they found that LSD1 depletion could induce transcriptional changes in neurodegenerative pathways as well as re-activation of stem cell genes in the degenerating hippocampus. Furthermore, the authors detected that LSD1 protein mislocalized in the cytoplasm associated with pathological protein aggregates in brains of AD patients. Although it remains unclear whether the functional impairment of LSD1 is directly connected with the neurodegeneration, the sustained amount of LSD1 in the brain is required to maintain the neuronal function (Christopher et al., 2017; Swahari and West, 2019). Noteworthy, several lines of studies have shown that specific pharmacological inhibition of LSD1 displayed significant neuroprotective effects in CNS disorders, underlying the therapeutic potential of LSD1 intervention in such diseases related to epigenetic dysregulation (Matsuda et al., 2019; Maes et al., 2020; Baba et al., 2021).

**FIGURE 1:**
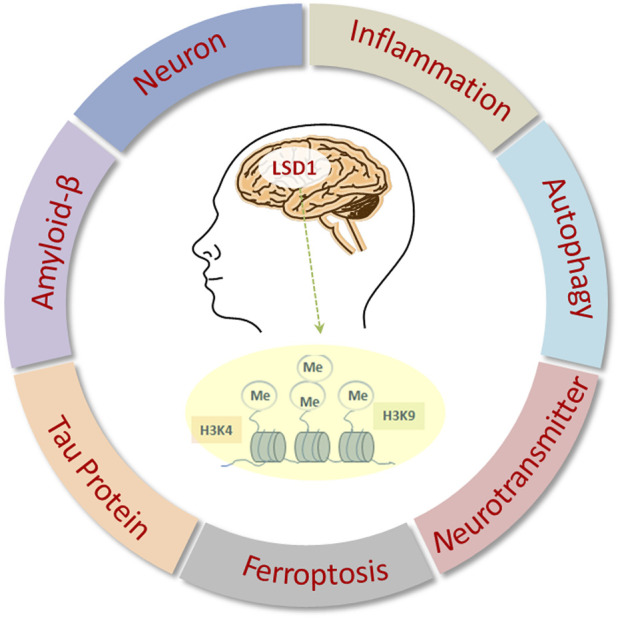
Biological functions of LSD1 in AD.

Here, this review focuses on the recent hallmark findings on the functional role of LSD1 in neurodegenerative disorders, particularly AD, along with its therapeutic potential in the treatment alternatives of AD (Figure 1).

## 2 Structure and modification function of lysine-specific demethylase 1

**FIGURE 2:**
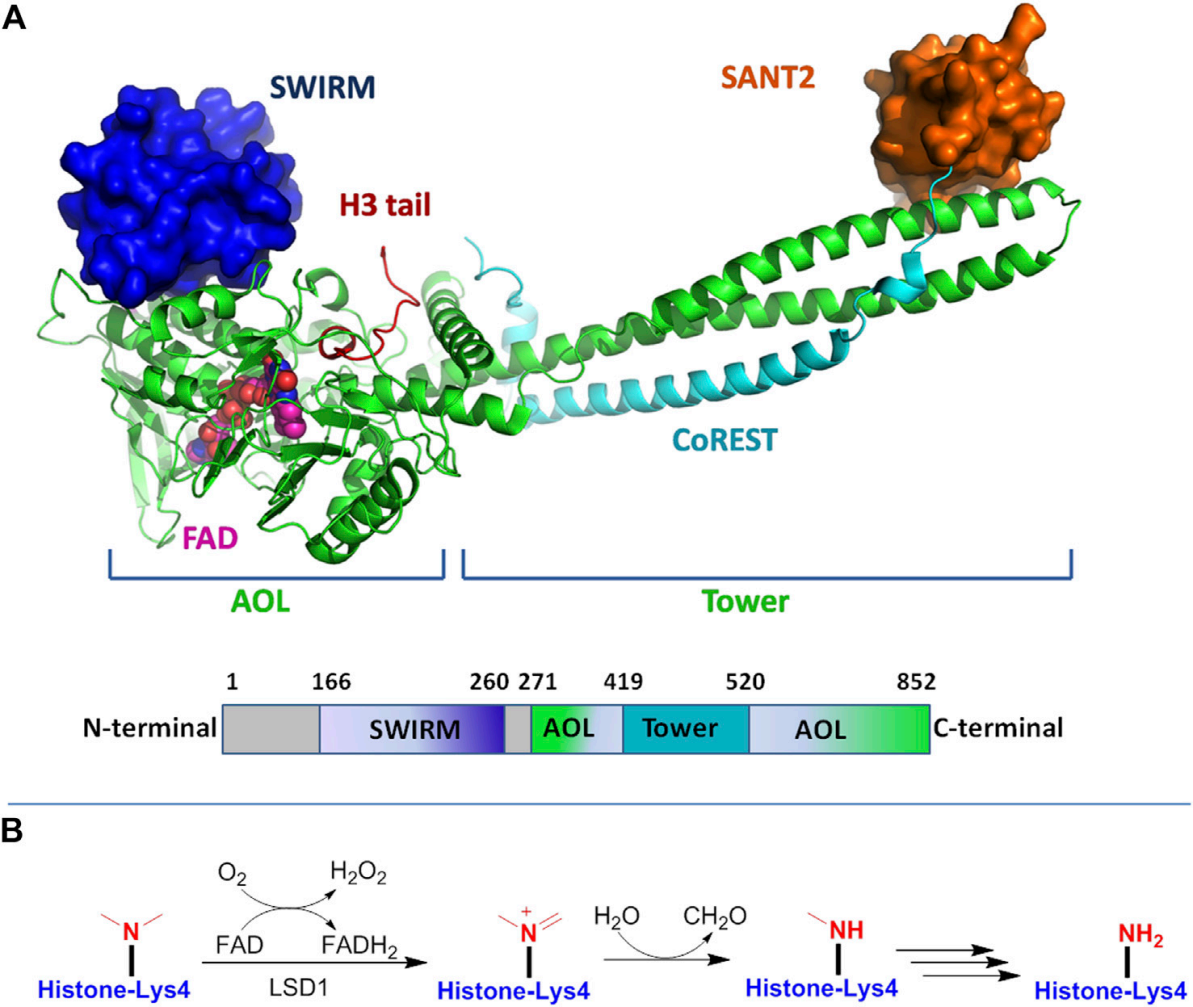
**(A)** Structure of LSD1 protein: up for the 3D model and down for the schematic illustration of LSD1 domains. Figures were created using PyMol (http://pymol.sourceforge.net); **(B)** FAD-dependent demethylation mechanism of mono-methylated lysine residue by LSD1.

LSD1 belongs to the FAD-dependent amine oxidase superfamily. The structure of LSD1 is highly conserved, and is divided into three major protein domains: an N-terminal SWIRM (Swi3p/Rsc8p/Morira) structural domain, a central protruding Tower domain, and a C-terminal amine oxidase like (AOL) domain (Figure 2A) (Chen et al., 2006).

The SWIRM domain interacts tightly with AOL domain to form a core structure, which covalently binds to FAD responsible for the enzymatic catalysis. The Tower domain protrudes from the AOL region, offering a binding platform to interaction with the LSD1 partners and CoREST. With FAD as coenzyme, LSD1 specifically catalyzes the demethylation process of mono- and di-methylated histone H3 lysine 4 (Shi et al., 2004). As shown in Figure 2B, in this catalytic reaction, the methyl lysine residue is oxidized by FAD to producing an imine intermediate, which is subsequently subjected to hydrolysis reaction to form a demethylated lysine and a formaldehyde molecule, while the reduced FAD is recovered by reoxidation. Due to the reaction requiring a hydride transfer or sing electron for the imine formation, LSD1 only enables the demethylation of mono-/di-methylated lysine, but not tri-methylated ones (Forneris et al., 2005; Chen et al., 2006; Forneris et al., 2008). LSD1 can also remove mono- and dimethylated K9 of histone 3 by forming complexes with androgen receptor or other nuclear receptors (Metzger et al., 2005; Garcia-Bassets et al., 2007). On the other hand, the demethylation activity of LSD1 may be negatively impacted by other histone modifications such as H3K9 deacetylation or H3S10 phosphorylation (Perillo et al., 2008; Binda et al., 2010). In addition to histone substrates, LSD1 could also demethylate nonhistone proteins such as p53, E2F transcription factor, DNA methyltransferases (DNMTs) and further modulate their activity (Ooi et al., 2007; Ciccone et al., 2009).

**FIGURE 3:**
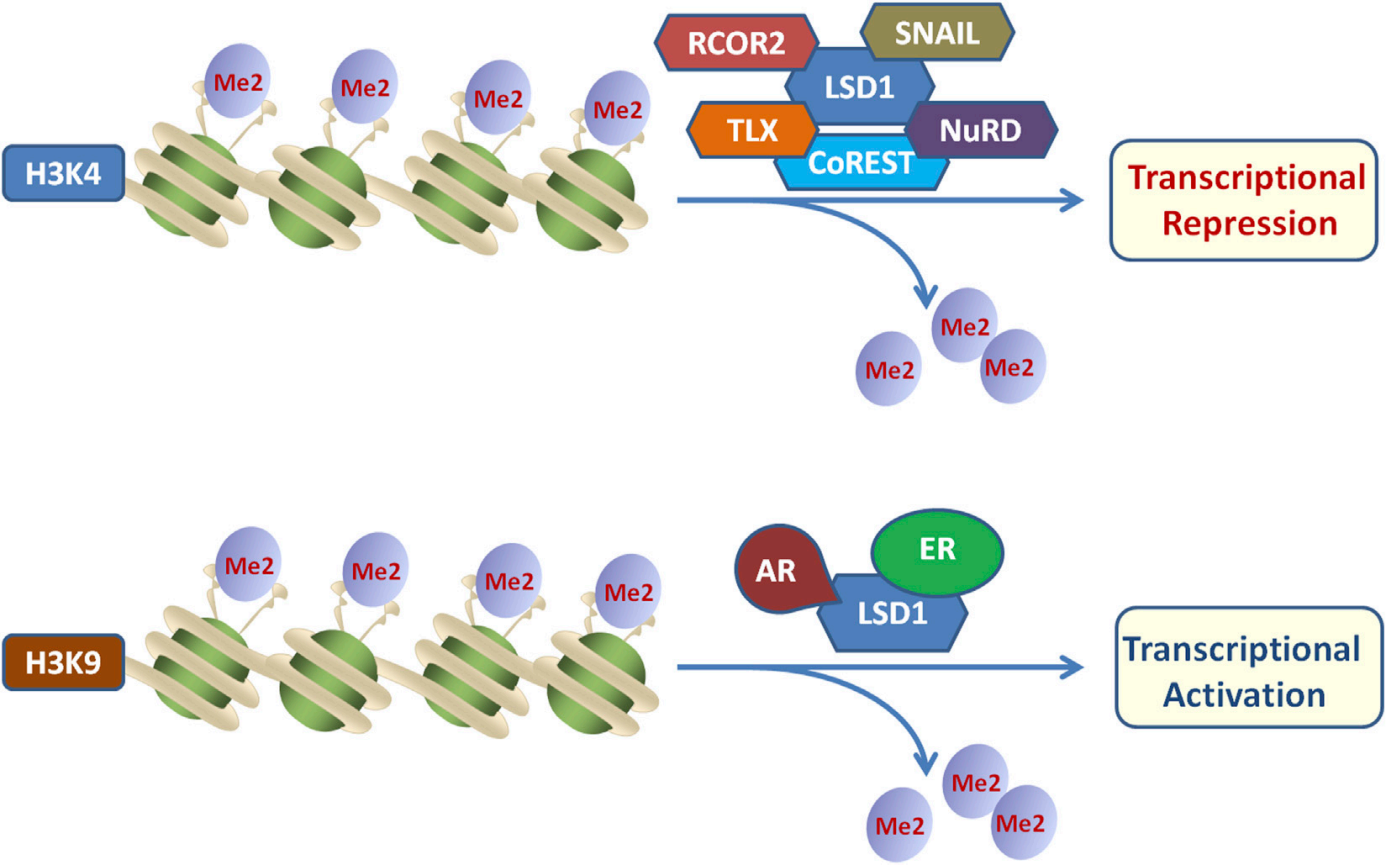
Dual functions of LSD1 as transcriptional repressor or activator by binding distinct cofactors.

Moreover, histone modification is usually associated with the activation or repression of gene transcription. LSD1 plays a repressive role in gene transcription through the interaction with CoREST, nucleosome remodeling and other deacetylase complex, and LSD1 is also capable of promoting transcriptional activation by binding to androgen receptor or estrogen receptor, thereby regulating gene expression (Figure 3) (Metzger et al., 2005). Besides, LSD1 is recruited by zinc finger transcription factors into repressive complexes to interact with RCOR and HDAC1/2 to demethylate H3K4me1/2 marks associated with the active transcription state (Barski et al., 2007; Upadhyay et al., 2014). By regulating the expression of target genes, LSD1 is closely related to tumorigenesis, pluripotent stem cells, and neurodegenerative diseases (Maes et al., 2015; Stefano et al., 2016).

## 3 Lysine-specific demethylase 1 mediates neuroinflammation in Alzheimer’s disease

Neuroinflammation is a defensive response of the immune system of the brain, protecting the CNS against infectious insults, injury and disease (Spencer et al., 2012; Zhang and Jiang, 2015). Current studies show that a significant portion of the occurrence and progression of AD is attributed to neuroinflammation (Culibrk and Hahn, 2020). Neuroinflammation is not a resultant role driven by emerging senile plaques and neurofibrillar tangles, but promotes the pathogenesis of AD as to the plaques and tangles themselves (Zhang et al., 2013). An analysis of clinical manifestation including mild cognitive impairments prior to dementia stage further supports the involvement of neuroinflammation in AD pathogenesis (Heneka et al., 2015). Neuroinflammation is a chronic response, during which microglia might have been initiated to cause a rapid switch to a damaging phenotype (Colton et al., 2006). Thus, neuroinflammation is usually found to occur at the early stage of AD progress prior to the presence of cognitive deterioration (Fu et al., 2019). In AD, microglia are able to bind to soluble Aβ oligomers and Aβ fibrils *via* cell surface receptors such as SCARA1, CD36, CD14 as well as Toll-like receptors, and these receptor engagement could induce the production of proinflammatory cytokines to partly contribute the inflammatory response (Liu et al., 2005; Stewart et al., 2010). Besides, accumulation of Aβ and neuronal debris could also activate inflammatory factors to stimulate chronic inflammation (Streit et al., 2009; Krabbe et al., 2013).

Emerging evidences have showed that histone modifications are closely associated with chromatin remodeling and gene transcription, and further play critical roles in regulating inflammation associated diseases (Sun et al., 2019; Surace and Hedrich, 2019). Kim et al. (2018) identified PKCα-LSD1-NF-κB axis as a new epigenetic pathway to activate and amplify the inflammation reaction, and pharmacological inhibition of LSD1 significantly reduced the excessive systemic inflammatory response. Zhuo et al. (2019) reported that inhibition of LSD1 could ameliorate Ox-LDL-stimulated NLRP3 activation and inflammation by promoting autophagy *via* PI3K/Akt/mTOR signaling pathway. In addition, LSD1 inactivation was also found to reduce inflammatory cell recruitment to tissues through mediating NF-κB signaling cascade and suppressing the production of cytokines (Wang et al., 2009). Lorenzo et al. (2021) found that the chromatin remodeling factor HMG20A mainly expressed in hypothalamic astrocytes, could affect the CNS development by inhibiting LSD1-CoREST complex, and inactivation of HMG20A may reduce neuroinflammation.

The current findings support neuroinflammation as a causal role in the pathogenesis of AD, however the identification of inflammatory biomarkers has not yet been established as a valuable indication for diagnosis of AD, and it is supposed to be the focus of future studies (Heneka et al., 2015).

## 4 Lysine-specific demethylase 1 mediates autophagy

Autophagy is a natural and evolutionarily conserved catabolic process through a lysosome dependent regulated mechanism, which degrades the damaged and dysfunctional components and protein aggregates (Klionsky, 2008; Vijayakumar and Cho, 2019). Autophagy plays an important cytoprotective role in responding to the environmental change, and has a tight connection with cell survival and cellular homeostasis (Yu et al., 2018). The autophagy deficiency is closely associated with multiple kinds of human diseases such as caner and CNS diseases, and the regulation of autophagy has emerged as a potential strategy for treating these diseases (Djajadikerta et al., 2020).

In AD, autophagy is a key modulator of Aβ production and clearance. The aberrant metabolism of Aβ peptides and tau proteins is found to be affected by autophagy, which can cause the accumulation and aggregation of toxic proteins to aggravate AD pathogenesis (Nilsson et al., 2013; Nilsson and Sarido, 2014). Autophagy is usually considered as a cytoplasmic event, increasing findings indicate that epigenetic regulation is also linked to the autophagy process. In particular, histone methylations have crucial roles in regulating autophagy at multiple levels, and is involved in the transcriptional control of multiple downstream effectors (Fullgrabe et al., 2014). Multiple lines of studies suggest that LSD1 is a negative regulator of genes required for autophagic process, and that LSD1 depletion or inhibition is able to induce autophagy in a range of human cell lines (Byun et al., 2017; Chao et al., 2017). Periz et al. (2015) found that LSD1 with ubiquitination factor E4B (UBE4B) exerted synergistic effect to increase the clearance of misfolded proteins through the control of the p53-dependent transcription program. Moreover, Ambrosio et al. (2017) reported that LSD1 inhibition could elevate the expression of SENE2 and consequent inhibition of mTORC1 to trigger the induction of autophagy.

## 5 Lysine-specific demethylase 1 and neurotransmitters

Neurotransmitters are a class of signaling molecules secreted by neurons to impact cells across synapses, and are essential for signal transduction within the whole nervous system (Erum et al., 2019; Stolero et al., 2021; Lourenco et al., 2021). Neurotransmitter is released from presynaptic neuron and bound to postsynaptic receptors such as G-protein coupled receptors or ligand-gated ion channels, subsequently priming the intracellular signaling pathway (Predescu et al., 2019; Boczek et al., 2021). Neurotransmitters including glutamate, GABA, acetylcholine, glycine and norepinephrine, play key roles in neuronal communications to maintain the balance between excitatory and inhibitory synaptic transmission, connecting with many human diseases and mental disorders (Erum et al., 2019; Kandimalla and Reddy, 2017; Reddy, 2017). For instance, in the CNS, glutamate with its receptors regulates the majority of excitatory neurotransmission and synaptic plasticity (Riedel, 2003). The dysfunction of the normal signaling pathway mediated by glutamate is closely involved in a variety of neuropathological disorders (Bleich et al., 2003).

LSD1 with its paralog LSD2 belongs to the FAD-dependent amine oxidase enzyme family comprising monoamine oxidases, which enable the metabolization of norepinephrine, related neurotransmitters as well as polyamine oxidases that metabolize spermidine and other alkylamines (Hou et al*.*, 2010; Edmondson et al., 2004). LSD1 structure contains an amine oxidase domain which recognizes a class of enzymes that may catalyze the FAD-dependent oxidation of amine substrates ranging from amino acids to aromatic neurotransmitters (Forneris et al., 2009). Compared to WT mice, Lim et al. (2017) found that the LSD1 knock-in mice has higher probability of presynaptic neurotransmitter release, without differences in amplitude between the genotypes. Moreover, Longaretti et al. (2020a) discovered that glutamatergic transmission could regulate the ratio of LSD1 and neuroLSD1 associated with a prototypic negative feedback mechanism, in which the activation of N-methyl-D-aspartate receptor induces downregulation of neurolLSD1 to further limit postsynaptic glutamate responses. In addition, they also found that the splicing mechanism in chronic social defeat stress, transiently affected the amount of LSD1/neuroLSD1 to undergo desensitization (Longaretti et al., 2020b).

## 6 Lysine-specific demethylase 1 stimulates ferroptosis

Ferroptosis is a newly identified mode of cell death dependent on iron and has distinct morphological and biochemical features different from cell apoptosis, necrosis, autophagy, etc., (Dixon et al., 2012; Xie et al., 2016; Weiland et al., 2019). Ferroptosis is initiated by the imbalance of redox homeostasis due to aberrant increase of iron-dependent ROS production, leading to the accumulation of iron ions, increase of lipid peroxidation products and depletion of glutathione (Dixon et al., 2012; Kraft et al., 2020; Chen et al., 2021). The accumulation of lipid peroxides is proposed to be one of the hallmark events in the initiation and progression of ferroptosis (Cheng and Li, 2007). Emerging studies have shown that ferroptosis is involved with the pathophysiological mechanism of cognitive dysfunction such as vascular dementia and senile dementia (Yan and Zhang, 2019; Derry et al., 2020). In brain, ferroptosis induced by depletion of neuronal glutathione peroxidease 4 (GPX4), could lead to cognitive impairment, while ferroptosis inhibition alleviates cognitive disorder (Hambright et al., 2017). A number of evidences have demonstrated that ferroptosis is closely associated with AD pathogenesis and development. In the AD brain, the accumulation of pathological neuronal iron largely contributes to the oxidative damage through the overproduction of free radicals (Smith et al., 1997). In addition, ferroptosis also mediates the cytotoxicity of Aβ peptides, and the slow accumulation of Aβ peptides can cause the prolonged ferroptosis in neurons to induce additional toxicity (Huang et al., 2020). Although increasing findings have revealed the essential role of ferroptosis in the pathogenesis of AD, the exact mechanism of action of ferroptosis in AD remains unclear (Chen et al., 2021).

Feng et al. found that LSD1 could promote both ferroptosis and oxidative stress through the activation of TLR4/NOX4 pathway, and LSD1 inactivation could alleviate ferroptosis (Feng et al., 2022). In addition, Li et al. (2015) reported LSD1 inhibition could elevate the expression of antioxidant gene SLC7A11, an important ferroptosis regulator, which is crucial for GSH synthesis/release as well as confer potent GSH-dependent neuroprotective effects. The transcriptional factor Nrf2 is a crucial regulator factor of ferroptosis process, and the activation of NRF2-LSD1 complex can regulate downstream antioxidant gene expression (Lin et al., 2021). Overall, there is currently little research on molecular mechanism of LSD1-mediated ferroptosis, especially in AD field, and it is worthy of further investigation.

## 7 Lysine-specific demethylase 1 and tau protein

Hyperphosphorlation and aggregation of tau protein in brain are a well-known pathological hallmark of AD, which is associated with synaptic loss, neuroinflammation, and neuronal death. The aberrant tau phosphorylation disrupts its binding to microtubules to promote microtubule accumulation, leading to its aggregation into neurofibrillary tangles mostly known as a primary marker of AD (Cho and Johnson, 2003). Although a great progress has been made in the understanding of AD pathogenesis, the precise mechanism of how tau protein contributes to neurodegeneration remains poorly understood (Engstrom et al., 2020).

LSD1 is mainly localized in the nucleus, while pharmacological intervention could cause a translocation of LSD1 from the nucleus to the cytoplasm (Yabuta and Shidoji, 2019). In tau pathology, Christopher et al. (2017) found LSD1 is localized to tau aggregates and sequestered in the cytoplasm, and proposed that the reduction of LSD1 in nucleus impairs the survival of hippocampal and cortical. Engstrom et al. (2020) discovered that pathological tau protein excluded LSD1 from the nucleus in neurons to cause neuronal cell death, since nuclear LSD1 is required for neuronal survival. In addition, they also demonstrated that the reduction of LSD1 could accelerate the tauopathy phenotype, while the extra supply of LSD1 slowed down the process of neuronal cell death, indicating LSD1 as a critical mediator of pathological tau-induced neurodegeneration (Engstrom et al., 2020). Thus LSD1 could be a potential therapeutic strategy for AD treatment.

## 8 Lysine-specific demethylase 1 is required for neuronal development

LSD1 plays an essential regulatory role in neural stem cell proliferation and neuronal development, and is continuously required for neuronal progenitor cell maintenance and terminal differentiation (Sun et al., 2010; Zibetti et al., 2010; Zhang et al., 2014). LSD1 is recruited by orphan nuclear receptor TLX, essential for neuronal stem cell, to the promoters of its target genes for transcriptional repression, thereby promoting neural stem cell growth (Yokoyama et al., 2008; Sun et al., 2010). In addition, Zhang et al. (2014) reported that LSD1 is essential for neuronal progenitor cell maintenance during cortical development, through modulating H3K4 methylation of the LSD1-binding site downstream of atrophin 1. Genetic or pharmacological intervention of LSD1 could cause the elevated methylation of this binding site as well as reduction of atrophin 1, thereby leading to neuronal progenitor cell differentiation (Zhang et al., 2014). Moreover, LSD1 degradation mediated by JADE-2, an E3 ubiquitin ligase, has a unique role in regulating neurogenesis and neural differentiation (Han et al., 2014). Christopher et al. (2017) reported that inducible depletion of LSD1 of adult mice mainly occurs in neurons, suggesting that neurons are possibly more vulnerable to LSD1 expression. They also found that LSD1 inhibition caused by pathological tau aggregates in aging neurons of AD could lead to neuronal cell death and dementia. Laurent et al. (2015) identified LSD1+8a, an isoform of LSD1 in neuronal cells, regulates H3K9me2 methylation in collaboration with supervillin as a cofactor, playing a unique role in neurodegeneration. Another isoform of LSD1, LSDn enables the regulation of H4K20 methylation relating to gene transcription for neuronal function, while depletion of LSDn could cause severe cognitive impairments (Wang et al., 2015).

## 9 Discrepancy between genetic depletion and pharmacological inhibition of lysine-specific demethylase 1 in Alzheimer’s disease

As aforementioned, many genetic evidences reveal that LSD1 has an important protective role in neurodegeneration. In contrast with genetic depletion of LSD1, pharmacological inhibition of LSD1 with specific LSD1 inhibitors produces completely different outcomes, showing a promising treatment potential in CNS diseases. Such that huge discrepancy mainly lies in the fact genetic intervention and a small molecule inhibitor can perturb a protein’s activity in different ways to further result in different or even opposite results about the protein’s biological function (Knight and Shokat, 2007). In most cases, intervention with small molecules do not affect the expression of the protein of interest but catalytic activity of the protein, while genetic intervention such as gene knockout could lead to complete loss of the protein to further cause protein complex dissociation to cause the release of cofactors to activate other signal pathways (Knight et al., 2007). In terms of LSD1, a number of findings have shown that LSD1 depletion can cause embryonic lethality, and non-specific LSD1 inhibitors may lead to severe hematological toxicity due to the dissociation of LSD1-contained complex (Foster et al., 2010; Sprussel et al., 2012). For example, LSD1 in complex with grow factor-independent 1B (GFI1B), is reported to be responsible for hematopoietic cell development and differentiation, and LSD1 knockdown causes derepression of GFI1B-mediated genes to lead to hematopoietic stem cell expansion and abnormal hematological differentiation (Saleque et al., 2007; Sprussel et al., 2012; Ishikawa et al., 2017).

**FIGURE 4:**
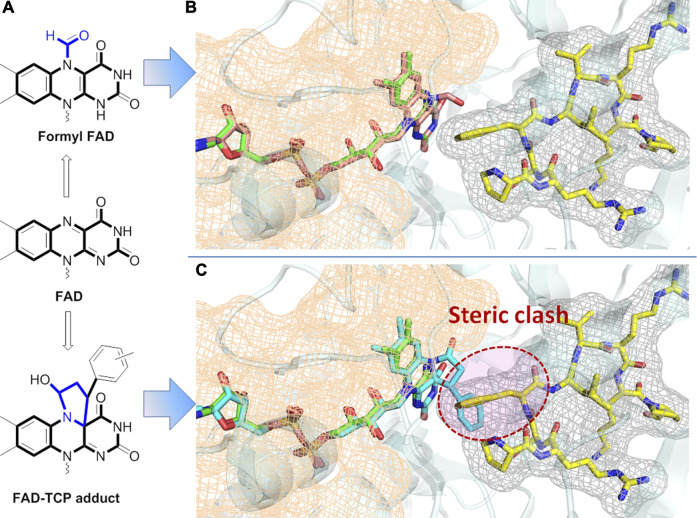
**(A)** Chemical structures of adducts of FAD with TCP in two different ways; **(B)** Superimposed structure of formyl FAD in LSD1 with GFI1B peptide (PDB: 7E0G); **(C)** Superimposed structure of FAD-TCP in LSD1 with GFI1B peptide (PDB:2Z5U). FAD is presented as stick model in green, formyl FAD in brown **(B)** and FAD-TCP adduct in cyan **(C)**.

On the other hand, tranylcypromine (TCP)-derived irreversible LSD1 inhibitors have been reported to exert hematological toxicities, probably due to the formation of TCP-FAD adduct to disrupt the interaction between LSD1 and cofactors (Maiques-Diaz et al., 2018; Matsuda et al., 2019). To address this issue, the researchers of Takeda Pharmaceutical Company have reported that their TCP-like LSD1 inhibitors such as TAK-448 and TAK-418 could normalize the aberrant epigenetic control of gene expression and efficiently improve cognitive deficits in animal models of CNS disorders, without causing hematological toxicity *in vivo* (Matsuda et al., 2019; Baba et al., 2021; Baba et al., 2022). Further investigation showed that both LSD1 inhibitors mechanistically formed unique compact formly FAD adduct (Figure 4), which posed the minimal impacts on LSD1-GFI1B complex. ORY-2001 of Oryzon Genomics is a brain penetrant LSD1 inhibitor with dual LSD1/MAO-B inhibition, and it is able to inactivate LSD1 in brain to improve memory deficit, behavior abnormalities and social obstacles of SMAP8 mice model (Maes et al., 2020). Overall, the alternation of the interactions of LSD1 with its cofactors is a highly interesting issue to address in terms of CNS field, and the role of LSD1 in different tissues and cell context also needs to be further elucidated (Maiques-Diaz and Somerrvaille, 2016).

## 10 Lysine-specific demethylase 1 inhibitors under clinical assessments

To date, many findings have shed light on the importance of LSD1 in various human diseases such as cancers and neurodegeneration, and targeting LSD1 has already become a powerful and promising therapeutic approach for such diseases. A lot of efforts have been made towards the development of potent and specific pharmacological inhibitors of LSD1 over the past decade.

**FIGURE 5:**
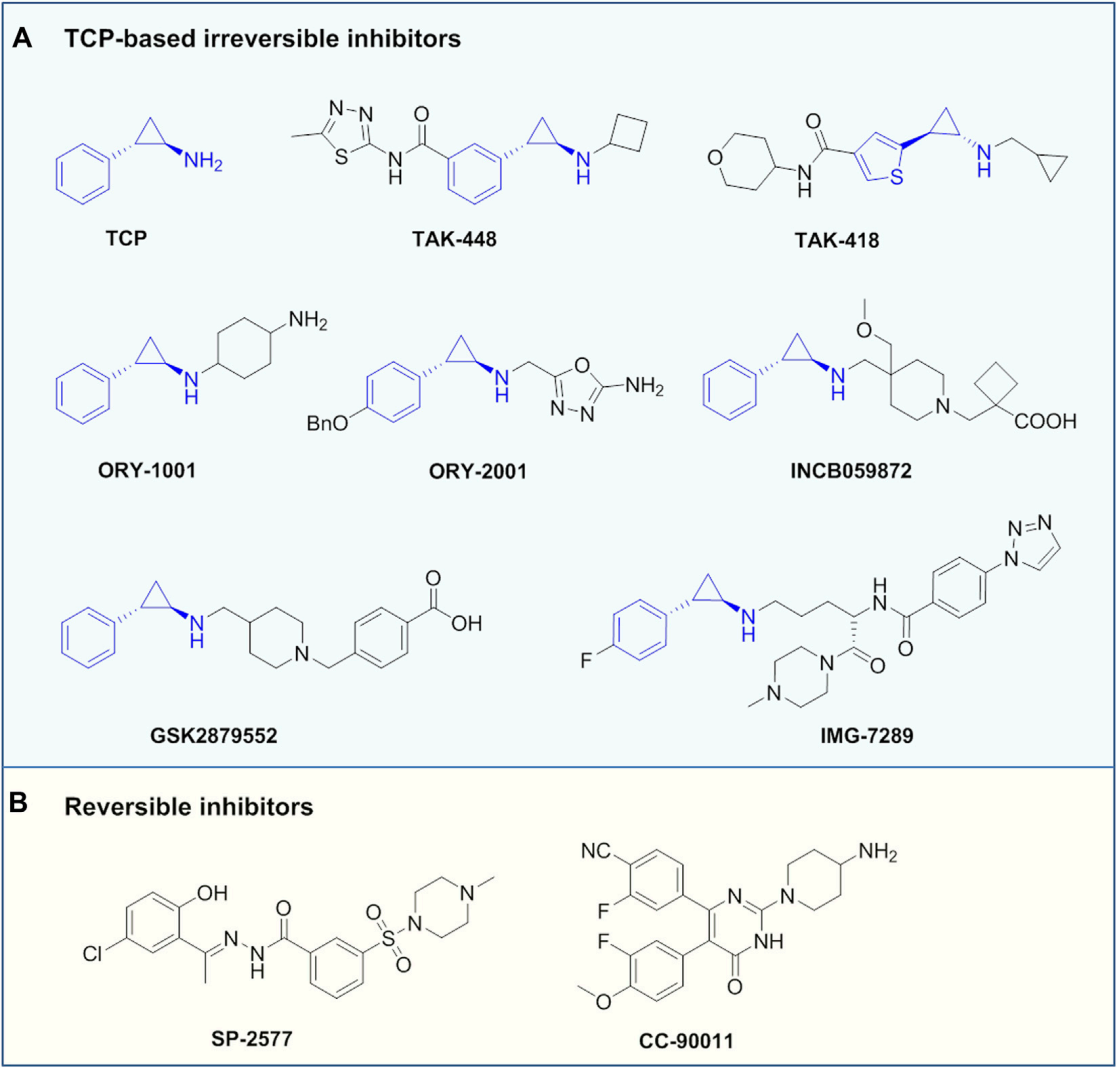
Representative LSD1 inhibitors under clinical assessment.

TCP is the first identified LSD1 inhibitor with weak inhibitory activity and poor specificity, which abrogates the catalytic activity by forming covalent adduct with the coenzyme FAD (Schmidt and McCafferty, 2007; Binada et al., 2010). Despite its insufficient activity, TCP has opened a path for the development of mechanism-based irreversible LSD1 inhibitors, making TCP an attractive chemical starting point for designing such inhibitors (Yang et al., 2015). Up to now, a large number of TCP-derived LSD1inhibitors with improved affinity and specificity, have been well developed, and many of which have laid a solid foundation for in-depth biological studies on the LSD1 inhibition in various diseases (Mould et al., 2015). Currently, TCP-derived LSD1 inhibitors such as ORY-1001, IMG7289, GSK2879552 and INCB059872 (Figure 5A) alone or in combination with other agents such as all-trans retinoic acid (ATRA), cytarabine or azacitidine, etc., are undergoing clinical assessments mainly for treatment of leukemias and solid tumors (Lee et al., 2016; Somervaille et al., 2016; Zheng et al., 2016). Apart from irreversible LSD1 inhibitors, the development of reversible inhibitors has also greatly stimulated interest in this field, albeit with more challenging (Mould et al., 2015). Compared with irreversible inhibitors, the development of reversible inhibitors lags far behind, and there are currently only two compounds SP-2577 and CC-90011 in clinical trials for cancer treatment (Figure 5B).

In addition, TAK-418 and TAK-448 (Figure 5A) are highly enzyme-specific LSD1 inhibitors without impact on LSD1 complex, and both compounds are reported to show therapeutic potentials for cancer, AD, autism symptom disorder and Kabuki syndrome et al. (Baba et al., 2021; Yin et al., 2021; Baba et al., 2022). ORY-2001 (also known as Vafidemstat, Figure 5A) is being evaluated in clinical assessments for treating CNS diseases such as AD and borderline personality disorder. ORY-2001 is a highly potent and orally active dual LSD1/MAO-B inhibitor developed by Oryzon Genomics SA, of note, it is so far the only LSD1 inhibitor currently under clinical assessment for treating AD. Preclinical investigation of ORY-2001 showed that it could mitigate cognitive impairment and reduce neuroinflammation, as well as normalize genes related to cognitive function, neuroplasticity and memory (Maes et al., 2020). Of note is that the *in vivo* pharmacological activity of ORY-2001 was mainly due to its inhibition of LSD1, while MAO-B inhibition had a minor contribution (Maes et al., 2020). In animal models, ORY-2001 could restore memory deficit and reduce aggression, as well as improve social behavior. The first-in-human clinical assessment demonstrated that ORY-2001 was safe and well tolerated, and possessed good PK/PD profile with rapid oral absorption, long half-life of 22 h, moderate systemic accumulation (mean AUC ration <2) after 5 days of administration. In particular, ORY-2001 did not cause apparent hematological toxicity, and transient reversible thrombocytopenia was observed in the treatment at 4 mg (Fang et al., 2019). ORY-2001 is currently in phaseⅡclinical trial (NCT03867253) to test the safety and preliminary efficacy in mild to moderate AD patients, and part of the clinical results demonstrated that ORY-2001 significantly reduced neuroinflammation after 6 and 12 months of treatment (Cummings et al., 2022). A summary of LSD1 inhibitors in clinical trails is provided in Table 1.

**TABLE 1:** LSD1 inhibitors in clinical trials (ClinicalTrails.gov in May 2022).

Inhibitor	Type	Phase	Trial number	Condition/Participant number/Max dose	Status
TCP	Irreversible	I	NCT02273102	AML and MDS/17/45 mg	Completed
		I/II	NCT02261779	AML/16/80 mg	Unknown
		I/II	NCT02717884	Non-M3 AML/60/80 mg	Unknown
		IV	NCT01430455	Bipolar depression/7/-	Completed
ORY-1001	Irreversible	I	NCT02913443	Relapsed ED SCLC/18/1.9 mg	Completed
ORY-2001	Irreversible	II	NCT03867253	Mild to moderate AD/24/1.2 mg	Completed
		II	NCT04932291	BPD/156/-	Recruiting
TAK-448	Irreversible	II	NCT02369796	HH/15/3 μg	Terminated
		II	NCT02381288	Low testosterone/17/-	
TAK-418	Irreversible	I	NCT03501069	Healthy participants/40,32/-	Terminated
		I	NCT03228433		
GSK2879552	Irreversible	I/II	NCT02929498	MDS/7/-	Terminated
		I	NCT02034123	SCLC/29/-	
		I	NCT02177812	AML/41/-	
INCB059872	Irreversible	I	NCT03132324	Sickle cell disease/12/2 mg	Terminated
		I	NCT03514407	Sarcoma/25/-	
		I/II	NCT02712905	Solid tumors, hematologic malignancy/116/-	
		I/II	NCT02959437	Advanced solid tumors/70/-	
IMG-7289	Irreversible	II	NCT04254978	ET/70/-	Active
		II	NCT04262141	ET, PV/24/0.6 mg	Recruiting
		I	NCT02842827	AML, MDS/45/-	Completed
		II	NCT05223920	MPN/80/-	Recruiting
		I/II	NCT05191797	SCLC/34/-	Recruiting
SP-2577	Reversible	I	NCT03895684	Advanced solid tumors/23/-	Completed
		I	NCT03600649	Ewing-related sarcomas/50/-	Recruiting
		I/II	NCT05266196	Sarcomas/10/-	Enrolling
		I/II	NCT04734990	MDS, MML/44/-	Recruiting
CC-90011	Reversible	I	NCT02875223	Lymphoma, Non-Hodgkin/91/-	Recruiting
		I	NCT04748848	Leukemia/1/-	Completed
		I	NCT03850067	SCLC/90/60 mg	Recruiting
		I	NCT04628988	Prostate cancer/10/60 mg	Recruiting

AD, Acute myeloid leukemia; SCLC, Small cell lung cancer; MDS, Myelodysplastic syndrome; BPD, Borderline personality disorder; HH, Hypogonadotropic Hypogonadism; ET, Essential thrombocythemia; PV, Polycythemia vera; MPN, myeloproliferative neoplasm; MML, Myelomonocytic leukemia.

## 11 Summary and perspectives

AD represents a complex and inexorable neurodegenerative disease. Although considerable efforts have been made to treat AD, poor progress has been achieved regarding to new therapeutics over the past decade (Long and Holtzman, 2019; Yiannopoulou and Papagergiou, 2020). LSD1 is an important epigenetic regulator and has a versatile role in mammalian biology and diseases. LSD1-mediated histone demethylation is closely associated with the pathogenesis of neurodegenerative diseases. In AD, LSD1 plays an important role in neural stem cell proliferation, neuronal differentiation and neuroprotection, and management of LSD1 provides an additional opportunity for developing therapeutic approaches in AD. It is important to note that genetic depletion of LSD1 may cause the pathogenesis of neurodegenerative disease, while enzymatic activity-specific inhibition of LSD1 demonstrates potential therapeutics for neurodegenerative diseases. These opposite results could be attributed to enzyme activity and complex function affected in different ways (Knight et al., 2007). Specific inhibitors of LSD1 form unique adduct with cofactor FAD to reduce its catalytic activity without affecting enzyme expression, while LSD1 depletion by genetic methods disrupt the interaction of LSD1 with cofactors to further activate other signal pathways.

To date, a fair number of LSD1 inhibitors have been developed, and some of them are currently undergoing clinical evaluation for cancers and CNS diseases. Despite remarkable advances of irreversible TCP-based inhibitors, there is only ORY-2001 in clinical trial for AD therapy. Mechanistically, the chemical process of LSD1 inhibition by TCP involves a single electron reduction mechanism *via* a homolytic cleavage of cyclopropyl ring to generate different FAD adducts directly impacting LSD1 complex (Yang et al., 2007; Fang et al., 2019). However, the exact mechanism of ring cleavage of TCP to form FAD adducts needs to be further studied, which is important to design potent and specific inhibitors without disrupting the interaction of LSD1 with cofactors like GFI1B, thereby reducing blood toxicity (Maiques-Diaz and Somervaille, 2016; Matsuda et al., 2019). Besides, the mechanism of action of TCP-like LSD1 inhibitors should be further elucidated in order to develop novel chemical entities possessing increased affinity and enhanced specificity. In addition, the high-resolution crystal structure of LSD1 protein with ORY-2001 is believed to provide valuable guideline for developing enzyme activity-specific LSD1 inhibitors for AD treatment.

To sum up, we propose the following general directions to consider for AD research: A) Investigations of reversible LSD1 inhibitors in AD treatment will be highly interesting to provide new pharmacological phenotypes for AD treatment; B) The way of TCP to form FAD adduct should be further elucidated to develop new LSD1 inhibitors with high affinity and cell-context specificity; C) Dual/multiple-targeted LSD1 inhibitors is of interest to combat complicated disease like AD, since this strategy has been largely developed for cancer treatment; D) Genetic depletion of LSD1 yields completely different results in AD compared with that of pharmacological intervention, and thus the research model should be carefully evaluated.
